# An analysis of predictors for heavy alcohol drinking using nationally representative survey data in Japan

**DOI:** 10.1186/s12889-021-10382-y

**Published:** 2021-02-16

**Authors:** Tasuku Okui

**Affiliations:** Medical Information Center, Kyushu University Hospital, 3-1-1 Maidashi, Higashi-ku, Fukuoka, 812-8582 Japan

**Keywords:** Alcohol drinking, Cross-sectional studies, Health literacy, Japan, Socioeconomic factors

## Abstract

**Background:**

Predictors of heavy or moderate alcohol drinking behavior have not been investigated using recent nationally representative survey data in Japan. This study investigated the effects of the predictors of heavy and moderate alcohol drinking in Japan using nationally representative survey data.

**Methods:**

Anonymous data from the 2013 Comprehensive Survey of Living Conditions in Japan were used to compare the predictors of heavy and moderate drinkers with those who abstain. Anonymized data that are resampled from all the survey data from the Ministry of Health, Labour and Welfare were obtained. Age group, marital status, living arrangements, educational level, household income, smoking status, and employment type were used as the explanatory variables. In addition, the drinking status (i.e., heavy drinker, moderate drinker, or abstainer) was used as the outcome variable. A multinomial logistic regression model was used, and an analysis comparing heavy drinkers and abstainers, as well as moderate drinkers and abstainers, was conducted.

**Results:**

Moderate drinking was positively associated with high educational level or high household income for men and women, as well as married status for men. In addition, unemployment was found to be negatively associated with heavy drinking for men and women, and an unmarried status was also found to be negatively associated with heavy drinking for men. Moreover, lower educational levels and smoking prevalence were found to be associated with heavy drinking for men and women. Furthermore, living alone for men and working in a large-scale company for women were also found to be predictors of heavy drinking.

**Conclusions:**

The preventive measures for heavy drinking were suggested to be particularly needed for those with lower educational levels and smokers. A call for attention among men living alone and among female employees in large-scale workplaces is also needed.

## Background

Alcohol consumption is a common behavior when interacting with others in Japan as well as in other Asian countries [1]. The age-standardized prevalence has been shown to continuously decrease for men in the past 20 years although the prevalence of drinking alcohol in Japan is several times larger in men than in women [2]. On the other hand, a report mentioned that binge and heavy drinking have been observed particularly in younger generations in Japan [3]. Furthermore, heavy alcohol drinking is a risk factor for multiple types of diseases and is associated with liver cancer, metabolic syndrome, all-cause mortality, and more in Japan [4–6]. Moreover, most of the high-income countries incur approximately ≥1% of their gross domestic product as alcohol-attributable costs. The social cost associated with heavy drinking is huge [7]. Therefore, assessing the predictors of heavy drinking in Japan and taking preventive measures against high-risk groups would be better.

Socioeconomic factors are associated with heavy alcohol drinking in many countries, including Japan [8–11]. The positive association between heavy drinking and low educational level or income has been indicated in some countries, including England and Korea [8, 9]. Investigating the effects of these factors against heavy drinking using nationally representative survey data in Japan is meaningful, especially because previous studies investigating this association are few. A previous study of predictors was conducted using nationally representative data in 2001 in Japan [10]. In that study, women with the lowest household income were shown to be associated with excess alcohol drinking. However, the data used were from 2001. Thus, reexamining these predictors using more recent data is important. In addition, factors suggested to be significantly associated with heavy drinking in other recent studies (e.g., educational level or smoking prevalence) were not used in the study. Moreover, whether the association between household income and heavy drinking still exists after adjusting for other factors is uncertain. Furthermore, another epidemiological study that was conducted in Japan investigated the association between socioeconomic status and heavy drinking and reported that a low educational level was associated with heavy drinking for men [11]. However, study participants were recruited only from around Tokyo. Reexamining this association using nationally representative data in Japan would be crucial. The previous study also targeted only male participants and did not investigate these predictors for women. Moreover, identifying predictors for moderate drinkers would also be important to help understand current drinking behavior among the Japanese, while the predictors have not been investigated using nationally representative survey data in a previous study. Therefore, in the current study, the effects of the predictors of heavy and moderate alcohol drinking using the recent nationally representative survey data were examined.

## Methods

### Data

The data used were from the 2013 Comprehensive Survey of Living Conditions, a survey conducted annually to investigate the household and income status of Japanese citizens. Moreover, a survey for health and savings status is conducted every 3 years [12]. More than 5000 districts in Japan were the survey targets chosen by stratified random sampling. Furthermore, all households in the districts were subjected to the survey. The districts in which all questionnaires about income, health, savings, care, and household status were surveyed were sampled from the randomly sampled districts. In 2013, the number of responses (response rate) was 27,081 households (74.4%) [12]. Participants received questionnaires regarding health status or household information and responded to them. Anonymized data that were resampled from all the survey data were obtained from the Ministry of Health, Labour and Welfare after obtaining permission from the Ministry. Data were removed in advance by the Ministry of Health, Labour and Welfare when a possibility exists that participants could be identified from the content of their responses. These anonymized data have been used in several previous studies [10, 13–16]. Overall, the data of 6260 households and 16,261 individuals were obtained. Data regarding the gender, age group, marital status, living arrangements, educational level, household income, smoking status, drinking status, drinking amount, and employment type were used from the anonymized data. These predictors were selected because an association between these factors and drinking status has been previously indicated [8–11, 17]. Consequently, ethical approval was not needed for this study because anonymized national statistics data were used.

### Outcome variable

The attributes of nonheavy drinkers could be different between those who abstain (abstainers) and moderate drinkers. Therefore, drinking status was divided into three groups: heavy drinkers, moderate drinkers, and abstainers. Two questions were asked about drinking status in the survey questionnaire. The first question was “How many days do you drink alcoholic beverages in a week?” with answer choices of “every day,” “5–6 days per week,” “3–4 days per week,” “1–2 days per week,” “1–3 days per month,” “merely drink,” “stop drinking,” and “not drinking.” The second question was “How much do you drink per day in a drinking day in terms of *sake*,” with answer choices of “less than one gou (180 mL of *sake*),” “one gou or more and less than two gou,” “two gou or more and less than three gou,” “three gou or more and less than four gou,” “four gou or more and less than five gou,” and “five gou or more.” *Gou* is a familiar unit of alcohol beverage in Japan, and 180 mL of *sake* contains approximately 22 g of ethanol [18]. The survey questionnaire explained that 180 mL alcohol content of *sake* is equivalent to approximately 500 mL of beer, 135 mL of *shochu* whose alcohol content is 20%, 110 mL of *shochu* whose alcohol content is 25%, 80 mL of *shochu* whose alcohol content is 35%, 350 mL of *shochu* whose alcohol content is 7%, 60 mL of whiskey, and 240 mL of wine [12]. Although no conclusive definition of heavy drinking exists in Japan, drinking 2 gou of *sake* per day has been used as the definition of heavy drinking in previous studies [10, 11]. Also, drinking 2 gou of *sake* or more per day is considered to be associated with a higher risk of developing lifestyle-related diseases [19]. Therefore, in this study, those who drank alcohol every day and drank 2 gou or more at a time, those who drank alcohol 5–6 days per week and drank 3 gou or more at a time, and those who drank 3–4 days per week and drank 5 gou or more at a time were classified as heavy drinkers. In other words, a person was classified as a heavy drinker if the average drinking amount per day in a week exceeded at least 2 gou of *sake* per day. In addition, abstainers were defined as those who responded to the questionnaire that they rarely drink, quit drinking, or do not drink. Other respondents, except those whose drinking status was unknown, were classified as moderate drinkers.

### Explanatory variable

The respondents were classified into six groups based on 10-year age groups (20–29, 30–39, 40–49, 50–59, 60–69, and 70–79 years) for respondents between 20 and 79 years old. Alcohol drinking is legally permitted at 20 years old in Japan. Thus, subjects who were ≥ 20 years old were utilized. Four types of marital status (married, never-married, divorced, and widowed) were identified. The respondents who were divorced and widowed were grouped into a single status because the number of widowed participants was relatively small. The participants were classified into two statuses for living arrangements based on the number of household members: living alone and living with others. Furthermore, the six educational levels of the survey were classified into three statuses: *low* (elementary or junior high school), *middle* (high school and technical school), and *high* (2-year college, university, or graduate school). Those whose educational level was unknown were classified as *unknown*. Moreover, household income levels were classified into four levels based on quantile. Employment type was classified into six statuses: working for a large-scale company (> 1000 employees or in a government or municipal office), working for a medium-scale company (30–1000 employees), working for a small-scale company (< 30 employees), self-employed, others (company officer, fixed-term employee, and so on), and unemployed. Smoking status was classified into two statuses: smokers (smoking every day or sometimes) and nonsmokers (former smoker or never smoker). Employment status (proper employee, temporary staff, and so on) was also asked in the survey questionnaire, although it was asked only on a fraction of the workers and was not used in this study.

### Statistical analysis

Participants were removed from the study if their anonymized data on working, alcohol drinking, or smoking statuses were unavailable because these variables were essential for the analysis. Moreover, participants between 20 and 79 years old were used for the analysis. A multinomial logistic regression model was used, and an analysis comparing heavy drinkers and abstainers (reference), as well as moderate drinkers and abstainers (reference), was conducted. A multivariate analysis using all the explanatory variables was conducted, and the adjusted odds ratio of each explanatory variable was calculated. The association between drinking status and socioeconomic factors was difficult to examine in older individuals because older individuals tend to be unemployed compared with younger people. Therefore, a subgroup analysis was also conducted targeting participants between 20 and 59 years old in addition to the analysis of all participants as was also done in a previous study [10]. All statistical analysis was conducted using R 3.6.3 (https://cran.r-project.org/).

## Results

**Fig. 1:**
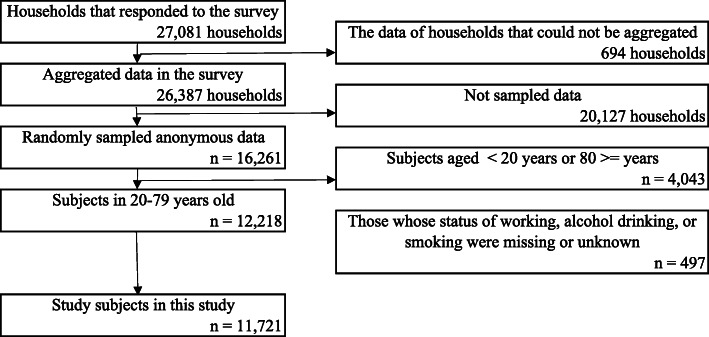
Flowchart of selecting the study subjects

Figure 1 indicates the flowchart of the selection of the subjects. The data of 11,721 individuals were used for the analysis after removing the data of participants whose working, smoking, or alcohol drinking statuses were unavailable and restricting subjects to those between 20 and 79 years old.

Table 1 shows the basic characteristics of the participants for each drinking status and gender. The drinking rates of younger and older ages tended to be smaller than those of middle-aged participants for both men and women. Married individuals comprised the majority in terms of marital statuses. Moreover, most of the participants were living with others. Heavy drinkers in women tended to have a lower educational level than the other drinking statuses. Moreover, no large differences exist in household income quantile among the drinking statuses. The unemployment rate tended to be higher in abstainers than in the other employment types. Furthermore, the prevalence of smoking was higher among heavy drinkers than the other drinking statuses.

**Table 1:** Basic characteristics of participants for each drinking status and sex

	Men			Women		
	Heavy drinker (*N* = 703)	Moderate drinker (*N* = 2763)	Abstainer (*N* = 2151)	Heavy drinker (*N* = 139)	Moderate drinker (*N* = 1703)	Abstainer (*N* = 4262)
Age						
20-29 years	15 (2.1)	281 (10.2)	322 (15.0)	9 (6.5)	202 (11.9)	459 (10.8)
30-39 years	118 (16.8)	412 (14.9)	382 (17.8)	29 (20.9)	282 (16.6)	675 (15.8)
40-49 years	151 (21.5)	473 (17.1)	373 (17.3)	51 (36.7)	385 (22.6)	639 (15.0)
50-59 years	170 (24.2)	531 (19.2)	275 (12.8)	27 (19.4)	352 (20.7)	687 (16.1)
60-69 years	191 (27.2)	634 (22.9)	450 (20.9)	21 (15.1)	324 (19.0)	986 (23.1)
70-79 years	58 (8.3)	432 (15.6)	349 (16.2)	2 (1.4)	158 (9.3)	816 (19.1)
Marital status						
Married	560 (79.7)	2066 (74.8)	1327 (61.7)	104 (74.8)	1128 (66.2)	2899 (68.0)
Never-married	97 (13.8)	548 (19.8)	685 (31.8)	16 (11.5)	337 (19.8)	711 (16.7)
Divorced or Widowed	46 (6.5)	149 (5.4)	139 (6.5)	19 (13.7)	238 (14.0)	652 (15.3)
Living arrangements						
Living with others	621 (88.3)	2530 (91.6)	1938 (90.1)	129 (92.8)	1552 (91.1)	3878 (91.0)
Living alone	82 (11.7)	233 (8.4)	213 (9.9)	10 (7.2)	151 (8.9)	384 (9.0)
Educational level^a^						
High	185 (26.3)	964 (34.9)	613 (28.5)	24 (17.3)	559 (32.8)	1042 (24.4)
Middle	112 (15.9)	299 (10.8)	305 (14.2)	9 (6.5)	137 (8.0)	599 (14.1)
Low	343 (48.8)	1270 (46.0)	1055 (49.0)	97 (69.8)	852 (50.0)	2255 (52.9)
Unknown	63 (9.0)	230 (8.3)	178 (8.3)	9 (6.5)	155 (9.1)	366 (8.6)
Household income quantile						
4th (highest) quantile	159 (22.6)	548 (19.8)	576 (26.8)	32 (23.0)	390 (22.9)	1200 (28.2)
3rd quantile	179 (25.5)	692 (25.0)	562 (26.1)	38 (27.3)	399 (23.4)	1061 (24.9)
2nd quantile	194 (27.6)	708 (25.6)	533 (24.8)	36 (25.9)	417 (24.5)	1021 (24.0)
1st (lowest) quantile	171 (24.3)	815 (29.5)	480 (22.3)	33 (23.7)	497 (29.2)	980 (23.0)
Employment type^b^						
Large-scale company	110 (15.6)	520 (18.8)	289 (13.4)	16 (11.5)	171 (10.0)	232 (5.4)
Middle-scale company	176 (25.0)	638 (23.1)	439 (20.4)	24 (17.3)	352 (20.7)	620 (14.5)
Small-scale company	88 (12.5)	297 (10.7)	244 (11.3)	15 (10.8)	208 (12.2)	381 (8.9)
Self-employed	116 (16.5)	328 (11.9)	265 (12.3)	18 (12.9)	111 (6.5)	317 (7.4)
Others	90 (12.8)	358 (13.0)	280 (13.0)	25 (18.0)	279 (16.4)	600 (14.1)
Unemployed	123 (17.5)	622 (22.5)	634 (29.5)	41 (29.5)	582 (34.2)	2112 (49.6)
Smoking status						
Non-smoker	337 (47.9)	1788 (64.7)	1499 (69.7)	84 (60.4)	1445 (84.9)	3888 (91.2)
Smoker	366 (52.1)	975 (35.3)	652 (30.3)	55 (39.6)	258 (15.1)	374 (8.8)

Each cell in the table indicates N (%)

^a^Low: elementary school/junior high school graduation; Middle: high school/technical professional school graduation; High: 2-year college/university graduation and more

^b^Small-scale company: employee of a company with fewer than 30 employees; Medium-scale company: employee in a company with 30 to 999 employees; Large-scale company: employee in a company with at least 1000 employees or government and municipal offices; Others: company officer and fixed-term employees etc.

Table 2 shows the results of multinomial logistic regression analysis for the predictors of heavy and moderate drinking among men. Heavy drinking was significantly associated with middle or older age. Consequently, the odds ratio was highest among those between 50 and 59 years old. Furthermore, moderate drinking was associated with older age compared with heavy drinking. Being married was also significantly associated with being a heavy drinker as was living alone. Moreover, low educational level was associated with heavy drinking, whereas middle- and low-educated individuals tended to be moderate drinkers than highly educated individuals. Furthermore, income was not associated with heavy drinking in the total population. However, lower-income groups tended to be abstainers rather than moderate drinkers more often than those in the high-income group. Furthermore, unemployed individuals were less likely to be heavy or moderate drinkers, but the reverse was true for smoking.

**Table 2:** Results of multinomial logistic regression analysis for the predictors of heavy and moderate drinking among men

	Total		20-59 years	
	Heavy drinker vs. Abstainer	Moderate drinker vs. Abstainer	Heavy drinker vs. Abstainer	Moderate drinker vs. Abstainer
	Adjusted OR (95% CI)	Adjusted OR (95% CI)	Adjusted OR (95% CI)	Adjusted OR (95% CI)
Age				
20-29 years	1.00	1.00	1.00	1.00
30-39 years	4.00 (2.25, 7.12)***	0.93 (0.74, 1.17)	3.84 (2.15, 6.86)***	0.92 (0.73, 1.16)
40-49 years	4.91 (2.76, 8.72)***	1.05 (0.83, 1.32)	4.65 (2.60, 8.31)***	1.02 (0.80, 1.30)
50-59 years	7.16 (4.00, 12.80)***	1.51 (1.17, 1.93)**	6.78 (3.77, 12.21)***	1.48 (1.15, 1.91)**
60-69 years	5.69 (3.14, 10.31)***	1.45 (1.12, 1.88)**		
70-79 years	2.65 (1.37, 5.14)**	1.59 (1.19, 2.14)**		
Marital status				
Married	1.00	1.00	1.00	1.00
Never-married	0.37 (0.28, 0.50)***	0.58 (0.49, 0.69)***	0.34 (0.25, 0.47)***	0.59 (0.48, 0.71)***
Divorced or Widowed	0.47 (0.31, 0.72)***	0.69 (0.52, 0.91)**	0.40 (0.22, 0.74)**	0.58 (0.38, 0.90)*
Living arrangements				
Living with others	1.00	1.00	1.00	1.00
Living alone	2.27 (1.57, 3.26)***	1.30 (1.02, 1.66)*	2.07 (1.31, 3.26)**	1.44 (1.07, 1.95)*
Educational level^a^				
High	1.00	1.00	1.00	1.00
Middle	1.01 (0.81, 1.26)	0.80 (0.69, 0.92)**	0.92 (0.71, 1.19)	0.73 (0.62, 0.87)***
Low	1.37 (1.01, 1.86)*	0.67 (0.54, 0.83)***	1.34 (0.86, 2.08)	0.48 (0.34, 0.69)***
Unknown	1.06 (0.75, 1.51)	0.84 (0.67, 1.06)	1.21 (0.77, 1.90)	0.87 (0.63, 1.19)
Household income quantile				
4th (highest) quantile	1.00	1.00	1.00	1.00
3rd quantile	0.97 (0.76, 1.25)	0.82 (0.70, 0.97)*	0.88 (0.65, 1.18)	0.77 (0.63, 0.93)**
2nd quantile	0.90 (0.69, 1.17)	0.80 (0.67, 0.95)**	0.81 (0.58, 1.12)	0.74 (0.60, 0.92)**
1st (lowest) quantile	0.80 (0.60, 1.08)	0.67 (0.55, 0.80)***	0.64 (0.44, 0.95)*	0.58 (0.45, 0.74)***
Employment type^b^				
Large-scale company	1.00	1.00	1.00	1.00
Middle-scale company	1.17 (0.87, 1.57)	0.93 (0.77, 1.14)	1.18 (0.86, 1.61)	0.95 (0.77, 1.17)
Small-scale company	1.02 (0.72, 1.46)	0.83 (0.66, 1.05)	1.06 (0.71, 1.57)	0.86 (0.66, 1.11)
Self-employed	1.11 (0.79, 1.57)	0.71 (0.56, 0.90)**	1.49 (0.98, 2.26)	0.98 (0.72, 1.33)
Others	0.87 (0.61, 1.24)	0.76 (0.60, 0.96)*	0.87 (0.57, 1.34)	0.85 (0.65, 1.11)
Unemployed	0.68 (0.47, 0.99)*	0.59 (0.46, 0.74)***	0.77 (0.45, 1.32)	0.49 (0.35, 0.67)***
Smoking status				
Non-smoker	1.00	1.00	1.00	1.00
Smoker	2.44 (2.03, 2.94)***	1.39 (1.22, 1.57)***	2.94 (2.33, 3.71)***	1.61 (1.37, 1.88)***

*p < 0.05, ** *p* < 0.01, *** *p* < 0.001; *OR* Odds ratio, *CI* Confidence interval

^a^Low: elementary school or junior high school graduation; Middle: high school or technical professional school graduation; High: 2-year college or university graduation and more

^b^Small-scale company: employees of a company with fewer than 30 employees; Medium-scale company: employees in a company with 30 to 999 employees; Large-scale company: employees in a company with at least 1000 employees or government and municipal offices; Others: company officer and fixed-term employees etc.

Table 3 shows the results of multinomial logistic regression analysis for the predictors of heavy and moderate drinking among women. Heavy and moderate drinking were significantly associated with age between 40 and 49 years, whereas those between 70 and 79 years old were less likely to be heavy drinkers. Moreover, marital status was not associated with heavy or moderate drinkers. The association between living arrangements and heavy drinking was not significant in women. In addition, middle educational level was associated with heavy drinking, whereas those with low or middle educational level tended to be more moderate drinkers than those individuals with higher education level. Income was not associated with heavy drinking rate in men. However, lower-income groups tended to be abstainers more than those in the highest-income group. In addition, working for a small- or middle-scale company was less likely to be associated with heavy drinking compared with working for a large company. A significant difference was observed between large- and middle-scale companies. Moreover, unemployed individuals were less likely to be heavy or moderate drinkers, whereas the reverse was true for smoking in men.

**Table 3:** Results of multinomial logistic regression analysis for the predictors of heavy and moderate drinking among women

	Total		20-59 years	
	Heavy drinker vs. Abstainer	Moderate drinker vs. Abstainer	Heavy drinker vs. Abstainer	Moderate drinker vs. Abstainer
	Adjusted OR (95% CI)	Adjusted OR (95% CI)	Adjusted OR (95% CI)	Adjusted OR (95% CI)
Age				
20-29 years	1.00	1.00	1.00	1.00
30-39 years	1.60 (0.71, 3.57)	0.98 (0.77, 1.24)	1.65 (0.74, 3.71)	0.95 (0.75, 1.21)
40-49 years	2.77 (1.26, 6.11)*	1.44 (1.13, 1.83)**	2.85 (1.28, 6.34)*	1.34 (1.05, 1.72)*
50-59 years	1.42 (0.61, 3.31)	1.27 (0.99, 1.64)	1.48 (0.63, 3.49)	1.16 (0.90, 1.50)
60-69 years	0.86 (0.36, 2.10)	1.08 (0.83, 1.40)		
70-79 years	0.12 (0.02, 0.61)*	0.74 (0.55, 1.00)*		
Marital status				
Married	1.00	1.00	1.00	1.00
Never-married	0.54 (0.29, 1.01)	0.99 (0.82, 1.21)	0.58 (0.31, 1.09)	0.91 (0.74, 1.12)
Divorced or Widowed	0.87 (0.48, 1.55)	1.08 (0.89, 1.31)	0.88 (0.45, 1.71)	1.30 (1.00, 1.68)
Living arrangements				
Living with others	1.00	1.00	1.00	1.00
Living alone	1.46 (0.67, 3.18)	1.25 (0.98, 1.60)	1.19 (0.43, 3.28)	1.68 (1.20, 2.34)**
Educational level^a^				
High	1.00	1.00	1.00	1.00
Middle	1.64 (1.02, 2.64)*	0.73 (0.64, 0.84)***	1.66 (1.00, 2.75)	0.76 (0.65, 0.89)***
Low	1.07 (0.46, 2.47)	0.60 (0.47, 0.76)***	1.98 (0.80, 4.91)	0.74 (0.49, 1.11)
Unknown	1.06 (0.48, 2.37)	0.91 (0.73, 1.14)	0.90 (0.35, 2.31)	0.87 (0.65, 1.16)
Household income quantile				
4th (highest) quantile	1.00	1.00	1.00	1.00
3rd quantile	0.96 (0.58, 1.56)	0.84 (0.72, 0.99)*	0.89 (0.53, 1.50)	0.86 (0.72, 1.03)
2nd quantile	1.13 (0.68, 1.86)	0.90 (0.76, 1.06)	1.01 (0.59, 1.74)	0.80 (0.66, 0.98)*
1st (lowest) quantile	0.97 (0.55, 1.69)	0.79 (0.66, 0.95)*	0.89 (0.48, 1.66)	0.75 (0.60, 0.95)*
Employment type^b^				
Large-scale company	1.00	1.00	1.00	1.00
Middle-scale company	0.48 (0.25, 0.95)*	0.82 (0.65, 1.05)	0.47 (0.24, 0.95)*	0.86 (0.67, 1.11)
Small-scale company	0.49 (0.23, 1.03)	0.82 (0.63, 1.07)	0.54 (0.25, 1.15)	0.88 (0.67, 1.17)
Self-employed	1.10 (0.53, 2.29)	0.61 (0.45, 0.83)**	1.09 (0.49, 2.46)	0.66 (0.45, 0.96)*
Others	0.57 (0.29, 1.12)	0.70 (0.54, 0.89)**	0.58 (0.29, 1.17)	0.72 (0.55, 0.94)*
Unemployed	0.48 (0.25, 0.90)*	0.51 (0.41, 0.65)***	0.50 (0.25, 0.97)*	0.52 (0.40, 0.67)***
Smoking status				
Non-smoker	1.00	1.00	1.00	1.00
Smoker	5.22 (3.56, 7.66)***	1.77 (1.48, 2.12)***	4.87 (3.21, 7.39)***	1.58 (1.29, 1.94)***

*p < 0.05, ** *p* < 0.01, *** *p* < 0.001; *OR,* Odds ratio, *CI* Confidence interval

^a^Low: elementary school or junior high school graduation; Middle: high school or technical professional school graduation; High: 2-year college or university graduation and more

^b^Small-scale company: employees of a company with fewer than 30 employees; Medium-scale company: employees in a company with 30 to 999 employees; Large-scale company: employees in a company with at least 1000 employees or government and municipal offices; Others: company officer and fixed-term employees etc.

## Discussion

This study analyzed the nationally representative survey data from 2013 and found that household income was not associated with heavy alcohol drinking when adjusting for other factors (e.g., educational level, smoking, company scale, and living arrangements) although these factors were shown to be associated with heavy drinking. Moreover, the association between each factor and heavy or moderate alcohol drinking was discussed below.

The prevalence of heavy and moderate alcohol drinking largely varied depending on age, particularly in men. The prevalence tended to increase with age for men and decrease from the middle ages for women according to a previous study on the prevalence of alcohol drinking in Japan [2]. This is consistent with the results of the current study. In addition, drinking prevalence was shown to decrease over the birth cohorts, particularly in men [2]. The increase of the prevalence of moderate drinking with age is considered to reflect the decrease of the prevalence over the cohorts. The peak of heavy alcohol drinking was the 50s and 40s for men and women, respectively. The age effect on alcohol drinking prevalence effects in this study reached its peak in men in their 50s although a recent report mentioned that binge and heavy episodic drinking were observed especially in younger generations in Japan [3]. Also, alcohol prevalence was decreasing over the cohorts in Japan, particularly in men [2]. Therefore, the middle age groups, in particular, is believed to need preventive measures against heavy alcohol drinking.

Never-married men were significantly less likely to be heavy alcohol drinkers than married men. In other countries, marriage has been shown to have a protective and harmful effect against alcohol abuse [9, 20]. However, the effect of marital status may have been confounded by living arrangements because many studies do not use living arrangements as an explanatory variable. The effect of living alone and being never-married on heavy drinking was inverse, as shown in the current study. Thus, treating these two factors independently is important, at least in Japan. Furthermore, the positive effects of marriage on health behavior and mortality have also been indicated in Japan [21]. However, not only the prevalence of heavy alcohol drinking but also that of moderate alcohol drinking was lower for never-married men compared with married men. Therefore, being married is believed to increase alcohol consumption in Japan, particularly for men.

Low educational level for men and middle educational level for women were significantly associated with heavy drinking. The prevalence of moderate drinking was significantly lower for individuals with these educational levels compared with higher educational levels. Consequently, alcohol abuse, but not alcohol consumption, was shown to be related to lower educational levels in this study. A previous regional study also showed the association between low educational background and heavy drinking in men [11] because education raises factual health-related knowledge and cognitive skills. Low educational level is also associated with other risky health behaviors in Japan [13, 14]. Therefore, health literacy is considered to mediate educational level and health behavior.

Household income was not associated with heavy alcohol drinking. A previous study in 2001 showed the association between the lowest income level and heavy alcohol drinking for women but did not consider educational level [10]. Consequently, a possibility exists that the preventive effect of high socioeconomic status on heavy drinking may be explained by educational level rather than household income. Another previous study conducted in recent years showed that higher income was significantly associated with heavy drinking [11]. The availability of enough money to purchase alcoholic beverages and work-related networking have been pointed out as possible reasons. Furthermore, the prevalence of moderate drinking was shown to be high among individuals with higher income for both men and women in the current study. Therefore, individuals with higher income have more opportunities and more time for drinking partly because drinking alcohol with colleagues from the workplace is a major social interaction behavior in Japan [11]. The results of employment type also showed that employees working for middle-scale companies were less likely to be heavy drinkers than those working for large-scale companies for women and that unemployed individuals were less likely to be heavy or moderate drinkers for both men and women. Historically, an increase in the employment rate of women was associated with an increase in the prevalence of alcohol drinking for women in Japan [22]. Women working for large-scale companies may have more opportunities to drink compared with women with other employment statuses.

Smoking prevalence was significantly associated with heavy drinking, regardless of gender and age subgroups. Smoking and alcohol dependence are known to co-occur, and several studies have shown the association between smoking prevalence and alcohol consumption [23–25]. Confounding factors may be associated with both behaviors although no causal relationship exists between smoking and alcohol consumption in other countries [24]. In particular, women living in urban were associated with smoking and heavy drinking in a previous study. Regional factors may be related to both behaviors [10]. Furthermore, smoking behavior was also shown to be associated with educational level in Japan [13, 14], and the co-use of smoking and heavy alcohol consumption more frequently exist in individuals with lower educational levels.

This study has some limitations. First, self-reported data were analyzed. Therefore, inaccurate responses were possible. In addition, answering patterns (e.g., missing drinking status) may be related to the predictors. Moreover, the regional data where each individual lived could not be used although a previous study inducted urban areas as a potential risk factor for excess alcohol drinking [10]. Furthermore, the heavy drinking definition used in the study is a kind of regular heavy drinking, which excludes episodic/irregular heavy drinkers. Additionally, heavy episodic heavy drinking (binge drinking) has also adverse effects on some diseases, including ischemic heart diseases [26]. Therefore, a study investigating the episodic heavy drinking predictors is also needed to reveal heavy drinker attributes. On the other hand, nationally representative survey data in Japan were used. Therefore, the results of this study are considered to be generalizable to all of Japan.

Heavy drinking was positively associated with smoking and lower educational background. Additionally, an association was also observed among men who were living alone. Some previous studies indicated the associations between each of these factors [27, 28], and it is believed that these factors are closely related. Health literacy was shown to be low in individuals with low socioeconomic status [29] and was considered to be related to multiple health behaviors. Thus, preventive measures for these groups of high-risk individuals are especially needed in Japanese public health. Furthermore, women working for large-scale companies tended to be heavy drinkers than those working for smaller companies. In addition, unemployment was negatively associated with heavy drinking. The drinking behaviors of women tended to be more influenced by working environments. Thus, attention should be given to women working for large-scale companies.

## Conclusion

The 2013 nationally representative survey data were used to investigate the effects of the possible predictors of heavy and moderate alcohol drinking. Consequently, lower educational levels and smoking prevalence were found to be positively associated with heavy drinking for men and women. Living alone for men and working in a large-scale company for women were also found to be predictors of heavy drinking. Moreover, unemployment was negatively associated with heavy drinking for men and women. Therefore, preventive measures for heavy drinking are needed for high-risk groups, such as smokers and those with lower educational backgrounds. Also, a call for attention among men living alone and among female employees in large-scale workplaces is needed.

## Data Availability

The anonymous data used in this study can be obtained from the Ministry of Health, Labour and Welfare in Japan. The statistical results obtained in this study were made and analyzed independently by the author using the anonymous data, and they are different from the statistics that the Ministry of Health, Labour, and Welfare made and published.
